# Breast cancer brain metastasis: from molecular insights to therapeutic innovation

**DOI:** 10.3389/fimmu.2026.1801614

**Published:** 2026-03-27

**Authors:** Xiaoxi Han, Wenjie Ma, Wenhui Zhao

**Affiliations:** Department of Medical Oncology, Harbin Medical University Cancer Hospital, Harbin Medical University, Harbin, China

**Keywords:** brain metastasis, breast cancer, molecular mechanism, prognosis, therapeutic targets

## Abstract

Breast cancer is the most common malignancy in women and a major cause of cancer-related mortality. While early-stage disease is often curable, many patients ultimately develop distant metastases, with the brain representing one of the most devastating sites. Breast cancer brain metastasis (BCBM) is particularly prevalent in human epidermal growth factor receptor 2 (HER2)-positive and triple-negative subtypes, leading to severe neurological symptoms, diminished quality of life, and poor prognosis. Despite progress in systemic therapy for primary tumors, outcomes for patients with BCBM remain poor, and these patients are frequently excluded from clinical trials. The pathogenesis of BCBM involves complex interactions between tumor cells and the central nervous system microenvironment. Crossing the blood-brain barrier and adapting to the brain niche requires tumor-stroma crosstalk, including signaling with astrocytes and microglia, which promotes immune evasion, therapeutic resistance, and metastatic outgrowth. Although advances in preclinical models and molecular profiling have provided valuable insights, critical mechanisms remain incompletely understood. Systemic therapies are increasingly important, with HER2-targeted agents, tyrosine kinase inhibitors, and subtype-specific regimens showing activity. Novel approaches, including poly (ADP-ribose) polymerase inhibitors, cyclin-dependent kinase 4/6 inhibitors, phosphatidylinositol 3-kinase inhibitors, and antibody-drug conjugates, are under evaluation. This review synthesizes epidemiology, molecular mechanisms, and emerging therapies of BCBM, underscoring advances achieved and highlighting the urgent need for novel targeted strategies and inclusive clinical trials.

## Introduction

Breast cancer (BC) represents the most frequently identified malignant tumor among women, with over 316,950 new cases annually in the United States, accounting for 32% of female cancer cases diagnosed. It is the second leading cause of cancer mortality, resulting in 42,170 deaths each year, and representing 14% of all cancer-related deaths in women (1). The lung, bone, brain and liver are the predominant metastatic sites of BC. Despite advances in early detection and therapeutic approaches, the incidence of metastatic BC continues to increase (2).

Breast cancer brain metastases (BCBM) constitute one of the most morbid and prognostically detrimental complications in the spectrum of advanced BC. BCBM impairs cognitive and sensory functions and is strongly associated with poor prognosis, leading to a markedly reduced quality of life (3). Current standard treatment for BCBM include neurosurgical resection, stereotactic or whole-brain radiation, chemotherapy or their combinations. Nevertheless, patient outcomes remain unsatisfactory due to restricted drug penetration across the blood-brain barrier (BBB) and intrinsic chemoresistance (4). Elucidating the underlying cellular and molecular mechanisms of BCBM is therefore crucial and may provide a foundation for improved prevention and therapeutic strategies.

While several prior reviews have addressed discrete aspects of BCBM biology and therapy (5–9), the present work seeks to move beyond pathway-centric perspectives by integrating tumor microenvironment signaling networks with an evidence-based appraisal of therapeutic strategies and their translational implications. Accordingly, this article is a narrative review that synthesizes current mechanistic and translational advances across the full metastatic cascade. We first delineate the early dissemination of tumor cells from the primary site and the systemic selection pressures that shape metastatic precursors during hematogenous transit. We then examine the molecular mechanisms underlying breast cancer cell extravasation across the BBB, followed by an analysis of the pathways governing subsequent brain colonization and dynamic tumor-brain crosstalk with resident neural and glial cell populations. We further interrogate immune modulation and metabolic reprogramming within the brain metastatic niche, before converging on tumor-intrinsic oncogenic signaling networks that sustain intracranial outgrowth. Finally, we provide a critical appraisal of emerging clinical strategies, elucidating how these mechanistic insights are catalyzing therapeutic innovation and informing next-generation treatment paradigms for BCBM.

## Literature search and selection strategy

A structured literature search was conducted using PubMed and Web of Science databases up to February 2026. Search terms included combinations of “breast cancer brain metastasis”, “blood-brain barrier”, “tumor microenvironment”, “immune modulation”, “targeted therapy”, and “clinical trial”. Priority was given to high-impact clinical studies, translational research, and mechanistic investigations relevant to intracranial progression. Additional references were identified through citation tracking of key publications.

## Clinical features and prognosis

BC is the most frequently diagnosed malignancy among women worldwide, accounting for approximately 2.3 million new cases annually. Despite substantial improvements in systemic disease control, brain metastases remain a common and clinically devastating complication. Population-based analyses indicate that 10-15% of patients with BC will develop clinically apparent brain metastases during the course of their disease, while autopsy series suggest a true incidence exceeding 30%, highlighting substantial underdiagnosis in routine clinical practice (10). The risk of brain metastasis is strongly influenced by molecular subtype, with cumulative incidence rates reported at 30–50% in HER2-positive disease and up to 40–46% in triple-negative breast cancer (TNBC), compared with less than 10% in hormone receptor (HR) positive, HER2 negative disease (11).

BCBMs are most often detected using computed tomography or contrast-enhanced magnetic resonance imaging (MRI), which can assess the size, number, and distribution of solitary or multiple lesions. These lesions have been identified in various regions of the brain, including the frontal, parietal, and occipital lobes, as well as the cerebellum and other brain regions. The most typical radiographic patterns include solid or rim enhancement, frequently accompanied by a central cystic non-enhancing core (6). The neurological symptoms of BCBMs arise primarily from tumor compression of brain tissue and increased intracranial pressure. Common manifestations encompass headache, vomiting, convulsions, nausea, seizures, dizziness, limb paralysis, visual impairment, and speech disturbances.

Despite advances in the management of primary BC, the overall prognosis for patients with BCBMs remains poor. Several prognostic indices have been developed to estimate survival. The graded prognostic assessment (GPA) is a relatively recent index incorporating clinical and pathological factors such as number of brain lesions, BC subtype, functional status, age at the time of brain metastasis diagnosis, and the presence of extracranial disease (12–14). Age at diagnosis represents an important demographic modifier of risk. Younger patients, particularly those diagnosed before the age of 50 years, demonstrate a disproportionately higher incidence of brain metastases, a pattern that parallels the increased prevalence of aggressive molecular subtypes in this population (15). Racial and ethnic disparities have also been consistently reported. Large registry-based studies from the United States show that Black women with BC have a higher likelihood of developing brain metastases and experience poorer survival after diagnosis compared with White women, even after adjustment for tumor subtype and stage, suggesting the contribution of structural inequities and differences in access to timely systemic and locoregional therapies (16). In addition, nomograms have been constructed to predict the likelihood of BCBM based on clinical and pathologic variables (17–20). Poor prognostic indicators include younger age, higher tumor grade, multiple metastatic sites, lung involvement, shorter disease-free survival, and negative hormone status (20–22). Conversely, infiltration of common myeloid progenitor (CMP) has been associated with less aggressive tumor biology, reduced risk of brain metastasis, and enhanced response to immunotherapy (23).

Beyond demographic factors, both non-modifiable and modifiable clinical risk factors shape the likelihood of brain metastasis. Non-modifiable factors include tumor biology, such as HER2 overexpression, basal-like transcriptional programs, and genomic instability, which promote central nervous system (CNS) tropism; germline pathogenic variants in BRCA1 and BRCA2, present in approximately 5-10% of BC cases, exemplify how inherited genomic instability concentrates this risk, with 44.7% of germline BRCA1 carriers experiencing distant recurrence subsequently developing parenchymal brain metastases (24). In contrast, modifiable factors relate primarily to disease control and surveillance. Poor extracranial disease control, delayed initiation of effective targeted therapies, and limited access to CNS imaging have all been associated with increased risk of symptomatic brain metastases (25). Together, these epidemiological and risk factor data underscore that BCBM arise from an interplay between tumor-intrinsic biology and patient-level determinants, reinforcing the need for risk-adapted monitoring strategies and earlier integration of CNS-directed considerations in high-risk populations.

Despite advances in systemic therapy and locoregional management, the prognosis of patients with BCBM remains poor and highly heterogeneous. Contemporary series report median overall survival ranging from 6 to 18 months, with outcomes largely dictated by molecular subtype, intracranial disease activity, and access to effective targeted therapies (26). Median intracranial progression-free survival after first local treatment typically remains limited to 4–8 months, and early intracranial progression frequently necessitates repeated radiotherapy or surgical intervention (25). Patients presenting with active or progressing brain metastases experience particularly adverse outcomes compared with those with stable, previously treated lesions (27). Together, these epidemiological and prognostic data highlight BCBM as a growing and clinically urgent problem driven by both tumor-intrinsic aggressiveness and patient-level factors.

## Mechanisms of breast cancer dissemination to the brain

### Early dissemination and microenvironment-driven intravasation

Metastatic colonization of the brain is initiated within the primary tumor through early dissemination and cellular plasticity. Epithelial-mesenchymal transition (EMT) playing a central role in enabling carcinoma cells to lose epithelial adhesion, acquire invasive and migratory phenotypes, thereby facilitating detachment and local invasion (28). EMT is driven by transcriptional programs that repress epithelial markers such as E-cadherin and promote mesenchymal traits including motility and resistance to apoptosis, allowing tumor cells to intravasate into the bloodstream or lymphatic system (28).

Dissemination is not solely tumor-cell intrinsic but is actively shaped by the tumor microenvironment (TME). Cancer-associated fibroblasts (CAFs) promote EMT through secretion of transforming growth factor beta (TGF-β) and other paracrine signals, driving downregulation of E-cadherin and induction of N-cadherin and vimentin (29, 30). CAFs further remodel the extracellular matrix (ECM) by releasing matrix metalloproteinases (MMPs), thereby degrading structural barriers and reducing cell-cell cohesion (31). Beyond structural remodeling, CAFs provide metabolic support by secreting lactate and glutamine, enhancing invasive capacity and stress tolerance (32, 33). The tumor microenvironment of metastasis (TMEM), characterized by coordinated interactions between tumor cells, macrophages, and endothelial cells, facilitates invadopodia formation and represents a key anatomical site for tumor cell intravasation (34–36). In parallel, chemokine signaling axes such as chemokine ligand C-X-C motif chemokine ligand 12 (CXCL12)- C-X-C motif chemokine receptor 4 (CXCR4) contribute to organ-directed migration, including tropism toward the brain (37).

### Systemic selection and survival of circulating tumour cells

Once in circulation, disseminated tumor cells encounter profound systemic bottlenecks, including hemodynamic shear forces, immune surveillance, and metabolic stress. Only a minority of circulating tumor cells (CTCs) survive these constraints. Selectins and integrins mediate transient vascular interactions, while platelet cloaking provides physical shielding and delivers pro-survival signals. Platelet-derived factors can further reinforce EMT and stem-like features, enhancing invasiveness and resistance to anoikis (38). Notably, CTCs may circulate as multicellular clusters with increased metastatic efficiency compared with single cells, reflecting cooperative survival advantages. In some contexts, stromal elements such as CAFs accompany tumor cells into circulation, providing additional matrix remodeling enzymes, growth factors including vascular endothelial growth factor (VEGF) and hepatocyte growth factor (HGF), and metabolic substrates that support stress adaptation (39, 40).

**Figure 1:**
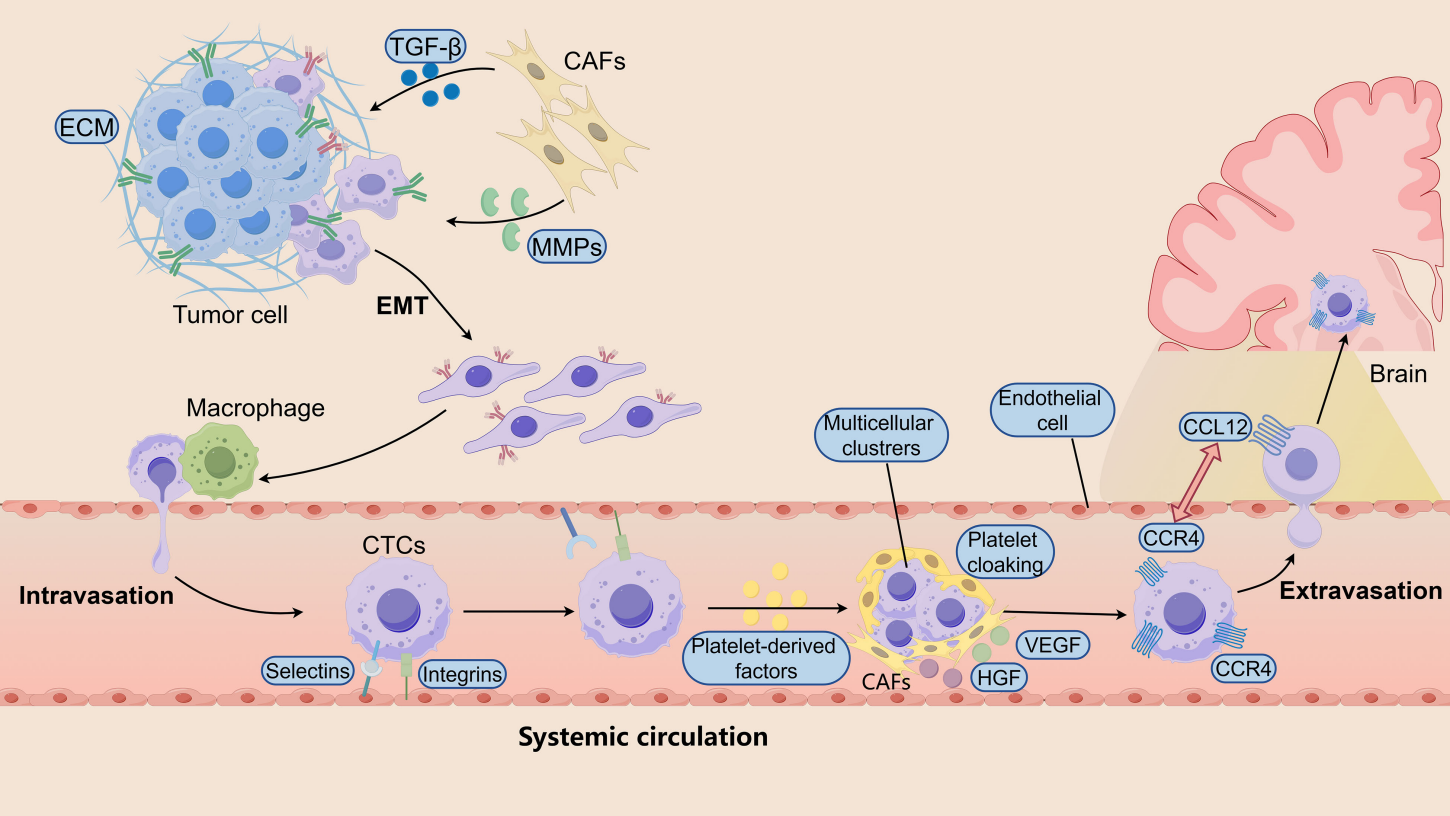
Early dissemination and systemic selection of metastatic precursors in BCBM. Metastatic colonization of the brain begins with early dissemination of tumor cells from the primary tumor. Within the primary tumor microenvironment, EMT enables tumor cells to acquire invasive and migratory phenotypes. CAFs promote EMT through secretion of TGF-β and matrix-remodeling enzymes such as MMPs, facilitating ECM remodeling and tumor cell detachment. Tumor-associated macrophages further support intravasation, allowing tumor cells to enter the circulation as CTCs. During systemic circulation, CTCs encounter immune and mechanical stresses. Selectins and integrins mediate transient interactions with endothelial cells, while platelet cloaking and platelet-derived factors enhance tumor cell survival. CTCs may also form multicellular clusters with increased metastatic potential. In addition, stromal components such as CAFs can accompany tumor cells and provide growth factors including VEGF and HGF. Chemokine signaling, particularly the CXCL12-CXCR4 axis, promotes brain tropism and facilitates tumor cell extravasation into the brain microenvironment.

Together, these processes indicate that early dissemination and systemic selection are governed by reciprocal tumor-microenvironment interactions (**Figure 1**). Brain metastasis therefore reflects the outcome of sequential selective pressures acting on plastic tumor cell populations rather than a singular event of vascular barrier crossing.

### Molecular mechanisms underlying blood-brain barrier traversal

#### The blood–brain barrier and blood-tumor barrier

BC cells give rise to three major types of brain metastasis depending on their anatomic location: parenchymal, leptomeningeal and choroid plexus metastases. Choroid plexus involvement is rare (41), leptomeningeal metastases account for approximately 8% of cases, whereas parenchymal metastases represent the most common form, comprising multiple lesions (78%) and solitary lesions (14%) (42).

The BBB, formed by continuous non-fenestrated capillaries, plays a pivotal role in regulating the entry of solute into the brain parenchyma and maintaining neuronal homoeostasis (43). Endothelial cells are connected by tight junction protein, including claudins, occludins, and intercellular adhesion molecules, which restrict paracellular diffusion (44, 45). Astrocytic foot processes provide additional structural and function support, linking neuronal activity to vascular regulation (46, 47). Astrocyte-derived factors are also critical for BBB development and maintenance (48). This highly specialized barrier preserves neurotransmitter compartmentalization and provides a stable microenvironment for neuronal signaling (43, 49, 50). When metastatic lesions form, BBB integrity is disrupted, giving rise to the blood-tumor barrier (BTB) (51).

The BTB, characterized by angiogenesis-driven vessels lacking tight junctions and astrocytic support, displays heterogeneous permeability and reduced blood flow (52–55). Increasing evidence indicates that the more clinically relevant challenge is the pronounced heterogeneity of the BTB. Rather than representing uniform BBB disruption, the BTB is a dynamic and spatially heterogeneous structure shaped by tumor-driven vascular remodeling with partial preservation of barrier function. As a result, drug accessibility varies substantially between patients and even among different regions within the same metastatic lesion, directly influencing therapeutic efficacy and resistance (51). In experimental models of BCBM, quantitative analysis of more than 2000 metastatic lesions showed that over 89% exhibited some degree of increased BTB permeability. Nevertheless, intratumoral concentrations of paclitaxel and doxorubicin generally remained below 15% of those achieved in extracranial tissues, and only about 10% of the most permeable lesions reached cytotoxic levels, highlighting a critical disconnect between radiographic permeability and effective drug exposure (51).

Clinical data further demonstrate that residual BTB function is influenced by molecular subtype and can be modulated by locoregional therapies. In patients with HER2-positive BCBM, pharmacokinetic analyses revealed a marked increase in cerebrospinal fluid exposure to trastuzumab following radiotherapy, with the serum-to-cerebrospinal fluid concentration ratio improving from 420 to 1 before treatment to 76 to 1 after radiotherapy (56). Consistently, immunohistochemical evaluation of resected brain metastases showed heterogeneous loss of barrier-associated proteins, including glucose transporter 1 and breast cancer resistance protein, with greater disruption observed in HER2-positive lesions than in triple-negative or basal-like subtypes (57). Moreover, dynamic contrast-enhanced MRI studies have demonstrated that radiotherapy induces time-dependent increases in BTB permeability. Lesions with low baseline permeability showed a marked rise in highly permeable tumor volume within weeks after treatment, suggesting a transient window during which intracranial drug delivery may be enhanced (58). Together, these findings underscore the importance of BTB heterogeneity as a determinant of intracranial drug efficacy and provide a mechanistic basis for optimizing the timing and integration of systemic therapies with locoregional treatment in BCBM.

#### Proteases and enzymes

MMPs involved in BBB disruption originate from both BC cells and cells of the neurovascular unit, with distinct but complementary roles in facilitating brain metastasis. Tumor-derived MMPs, particularly MMP2 and MMP9, are upregulated in invasive BC cells and directly degrade components of the vascular basement membrane and tight junction complexes, thereby promoting transendothelial migration and extravasation into the brain parenchyma. Experimental models have demonstrated that genetic or pharmacological inhibition of tumor-cell-derived MMP9 significantly reduces brain metastatic burden, supporting a causal role in early metastatic seeding (59).

In parallel, endothelial cells and reactive astrocytes within the brain microenvironment can be induced to express MMPs in response to tumor-derived cytokines and growth factors. Endothelial MMP activation contributes to the proteolytic cleavage of junctional proteins, including claudins and occludin, further compromising BBB integrity (60). This bidirectional protease activity establishes a permissive vascular niche in which tumor-intrinsic invasiveness and microenvironment-driven vascular remodeling act in concert to facilitate brain metastasis formation.

#### Secreted proteins and oncogenic drivers

Lipocalin-2 (LCN2) promotes proliferation, angiogenesis, and EMT, while cooperating with MMP-9 to remodel the ECM and impair BBB integrity (61). Ectonucleotide pyrophosphatase/phosphodiesterase 1 (ENPP1), secreted by HER2-positive BC cells, disrupts endothelial insulin signaling and the AKT/GSK3β/β-catenin pathway, leading to junctional protein loss and enhanced transmigration (62). Growth factor receptor-bound protein 2 (GRB2) drives HER2-positive BC brain metastasis through Ras/MAPK signaling and BBB penetration (63).

#### Extracellular vesicle–mediated vascular remodeling and permeability

Hypoxia within the primary tumor and metastatic microenvironment activates hypoxia-inducible factor (HIF) signaling in BC cells, which in turn promotes the selective loading of integrin β3 (ITGB3) into extracellular vesicles (EVs). These vesicles are subsequently taken up by endothelial cells, where ITGB3 activates vascular endothelial growth factor receptor 2 (VEGFR2) signaling and induces the expression of angiopoietin 2 (64). In conjunction with neuropeptides such as substance P (65)., this signaling cascade disrupts endothelial junctional proteins, including zonula occludens-1 (ZO-1) (66), thereby enhancing angiogenesis and increasing vascular permeability (67). Through this mechanism, hypoxia-driven extracellular vesicle signaling establishes a pro-permeability vascular state that facilitates tumor cell extravasation and supports the formation of brain metastases.

#### Non-coding RNAs and extracellular vesicles

Exosomal miR-105 disrupts ZO-1 and increases endothelial permeability (68). LncRNA GS1-600G8.5, carried in BCBM-derived exosomes, downregulates ZO-1, claudin-5 and N-cadherin, weakening the BBB (69). Migrasomes transporting activating transcription factor 6 (ATF6) induce endoplasmic reticulum stress in brain endothelial cells, resulting in the loss of ZO-1 and vascular endothelial cadherin (VE-cadherin) and facilitating tumor migration (70).

#### Other mediators of BBB transmigration

The epidermal growth factor receptor (EGFR) ligand heparin-binding EGF-like growth factor (HBEGF), cyclooxygenase-2 (COX-2), and ST6 sialyltransferase 5 (ST6GALNAC5) all promote BBB penetration, with ST6GALNAC5 conferring brain-specific tropism (71). Semaphorin 4D (SEMA4D) engages with Plexin-B1 receptors expressed on brain endothelial cells to enhance circulating tumor cell (CTCs) penetration (72). T lymphocytes upregulate guanylate-binding protein 1 (GBP1) in tumor cells, thereby promoting their transendothelial migration (73), while CAFs increase BBB permeability (74). Moreover, endothelial-mesenchymal transition has been implicated as a novel pathway enabling BBB transmigration (75).

**Figure 2:**
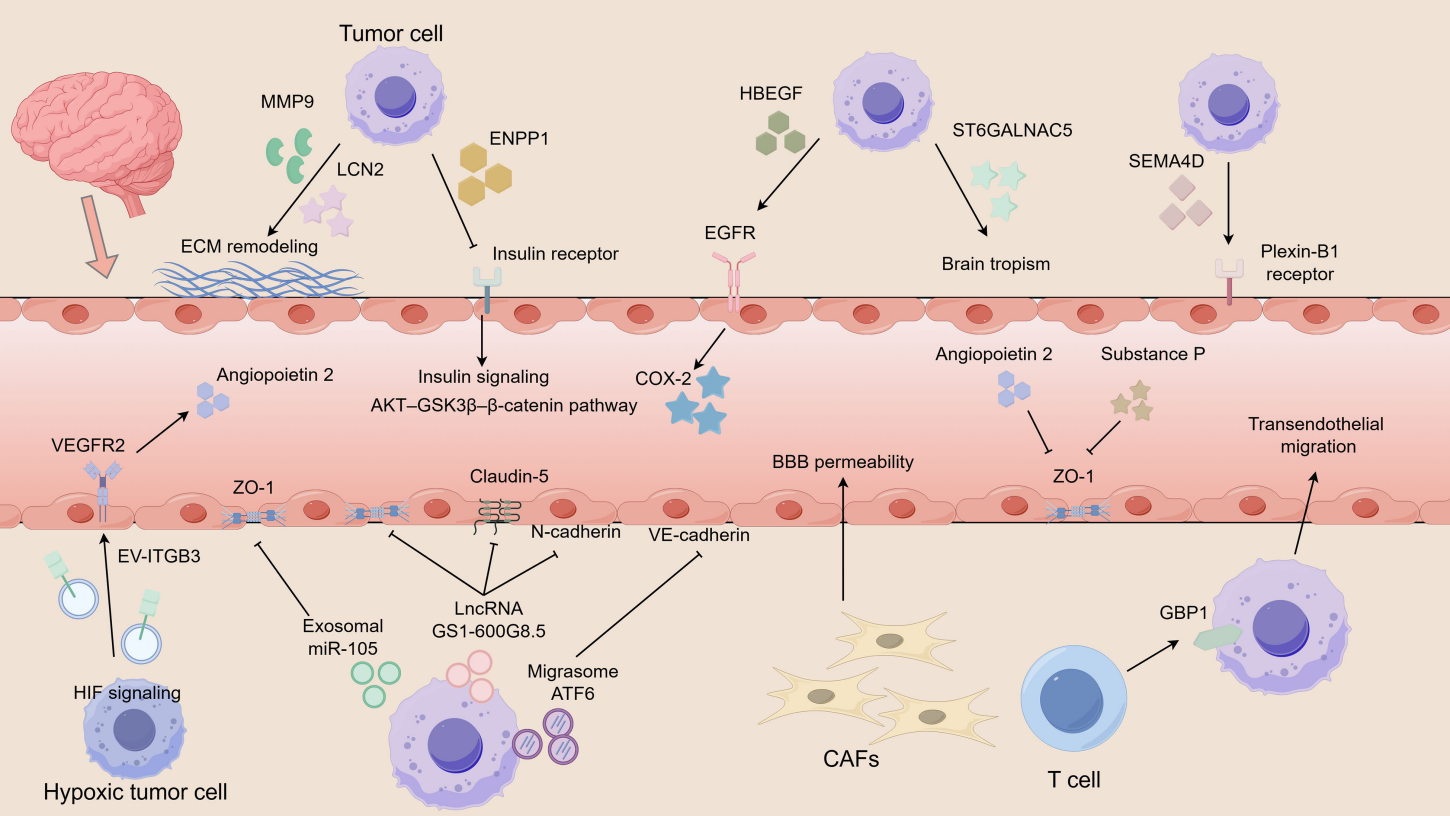
Molecular mechanisms promoting BBB disruption and transmigration in BCBM. Breast cancer cells disrupt the BBB through coordinated actions of secreted factors, EVs, and non coding RNAs. Tumor derived LCN2 cooperates with MMP9 to remodel the ECM and promote EMT. ENPP1 interferes with endothelial insulin signaling and alters the AKT-GSK3β-β catenin pathway, leading to junctional destabilization. Hypoxia driven EVs enriched in ITGB3 activate VEGFR2 signaling and induce angiopoietin 2 expression, promoting angiogenesis and vascular permeability. Exosomal miR105 and lncRNA GS1 600G8.5 disrupt tight junction proteins including ZO-1, claudin-5 and N cadherin, whereas migrasome mediated ATF6 signaling reduces VE cadherin expression. Additional mediators including HBEGF, ST6GALNAC5, SEMA4D, GBP1 and CAFs further facilitate tumor cell transmigration across the barrier and promote brain metastasis formation.

These findings demonstrate that BC cells exploit diverse molecular mechanisms, including angiogenic signaling, protease activity, secreted oncogenic proteins, non-coding RNAs, and interactions with stromal and immune cells, to compromise the integrity of the BBB and establish brain metastases (**Figure 2**). Despite their heterogeneity, these pathways converge on a common outcome: the disruption of endothelial junctions and increased vascular permeability, which together facilitate tumor cell transmigration. A deeper understanding of these mechanisms will be essential for developing targeted interventions aimed at preserving BBB integrity and preventing or treating BCBM.

## Brain colonization in breast cancer metastasis

### Dormancy and reactivation

After extravasation from the circulation, cancer cells must overcome host defenses to establish colonies. A substantial fraction of disseminated tumor cells undergoes apoptosis, while others enter a dormant state with the potential for recurrence, and only a minority immediately proliferate. Dormancy begins when p38 signaling is activated, leading to reduced extracellular signal-regulated kinase (ERK) activity. This dormant state ends once the ERK-to-p38 activity ratio increases, favoring ERK signaling (76). In a hydrogel-based model, the dormancy-associated proteins p21 and p27 were found mainly in the nucleus of dormant cells. However, in proliferative BCBM cells, they were located in the cytoplasm. These cyclin-dependent kinase inhibitors (CDK) are known to be regulated by ERK signaling (77). Dormant cells can be reawakened by various stimulations, including vascular cell adhesion molecule-1 (VCAM), transforming growth factor-β1 (TGF-β1), and periostin (POSTN), while ECM stiffness also facilitates escape from dormancy (78). In addition, mucin 5AC (MUC5AC) enhances tumor cell adhesion and migration through cMET/CD44v6 interactions, and inhibition with bozitinib has been shown to suppress brain colonization (79).

### Vascular co-option and early outgrowth

Actively proliferating metastatic cells often establish micrometastases by co-opting existing blood vessels, thereby preparing for sustained lesion growth (80). BCBM cells secrete chemokine ligand 2 (CCL2) to attract macrophages. These recruited macrophages subsequently produce interleukin-6 (IL-6) and oncostatin M (OSM). These cytokines induce the long non-coding RNA lnc-BM. Once upregulated, lnc-BM facilitates the process of vascular co-option by activating the Janus kinase 2 (JAK2)/signal transducer and activator of transcription 3 (STAT3)/intercellular cell adhesion molecule-1 (ICAM) signaling axis. Additionally, it contributes to the survival of metastatic cells by inhibiting apoptotic pathways (4). L1 cell adhesion molecule (L1CAM), found in both neuronal and cancer cells, supports the translocation of BCBM cells from blood vessels into brain tissue by promoting their passage across the vascular-parenchymal boundary. By activating downstream neuroserpin, L1CAM prevents astrocyte-mediated plasmin generation, a natural suppressor of brain metastases (81). Similarly, β1 integrin enhances vascular co-option by facilitating the attachment of BC cells to the vascular basement membrane (82).

### Metabolic adaptations to the brain microenvironment

For long-term colonization, tumor cells must reprogram their metabolism to adapt to the unique brain niche. Elevated fatty-acid-binding protein 7 (FABP7) enhances fatty acid utilization and glycolysis, supporting survival under hostile conditions through a combined “Warburg effect” and lipid metabolism (83). Under glucose deprivation, glucose-regulated protein 94 (GRP94) upregulation increases inhibitor of apoptosis proteins (IAPs) and B-cell lymphoma 2 (BCL2). This response also triggers autophagy, thereby promoting survival (84). In parallel, gluconeogenesis is fueled by glutamine and branched-chain amino acids (85). The family with sequence similarity 50 member A (FAM50A)- chromosome 9 open reading frame 78 (C9ORF78) complex enhances asparagine synthetase (ASNS) activity, increasing asparagine production and driving brain metastasis, highlighting a potential metabolic vulnerability (86). To mitigate reactive oxygen species (ROS), lymphoid enhancer-binding factor 1 (LEF1) upregulates glutathione production, shielding tumor cells from oxidative damage (87). BC cells secrete EVs-derived miR-199b-5p, which disrupts neuron-astrocyte metabolic coupling by targeting solute carrier transporters (SLC1A2, SLC38A2, SLC16A7). This leads to extracellular retention of glutamate, glutamine, and lactate, fueling tumor growth and facilitating brain colonization (88). Retinoic acid receptor responder 2 (RARRES2) deficiency activates the phosphatase and tensin homolog (PTEN)/mammalian target of rapamycin (mTOR)/sterol regulatory element binding protein-1 (SREBP1) pathway, increasing glycerophospholipids and reducing triacylglycerols, thereby enhancing tumor cell survival and adaptation in the brain microenvironment (89).

**Figure 3:**
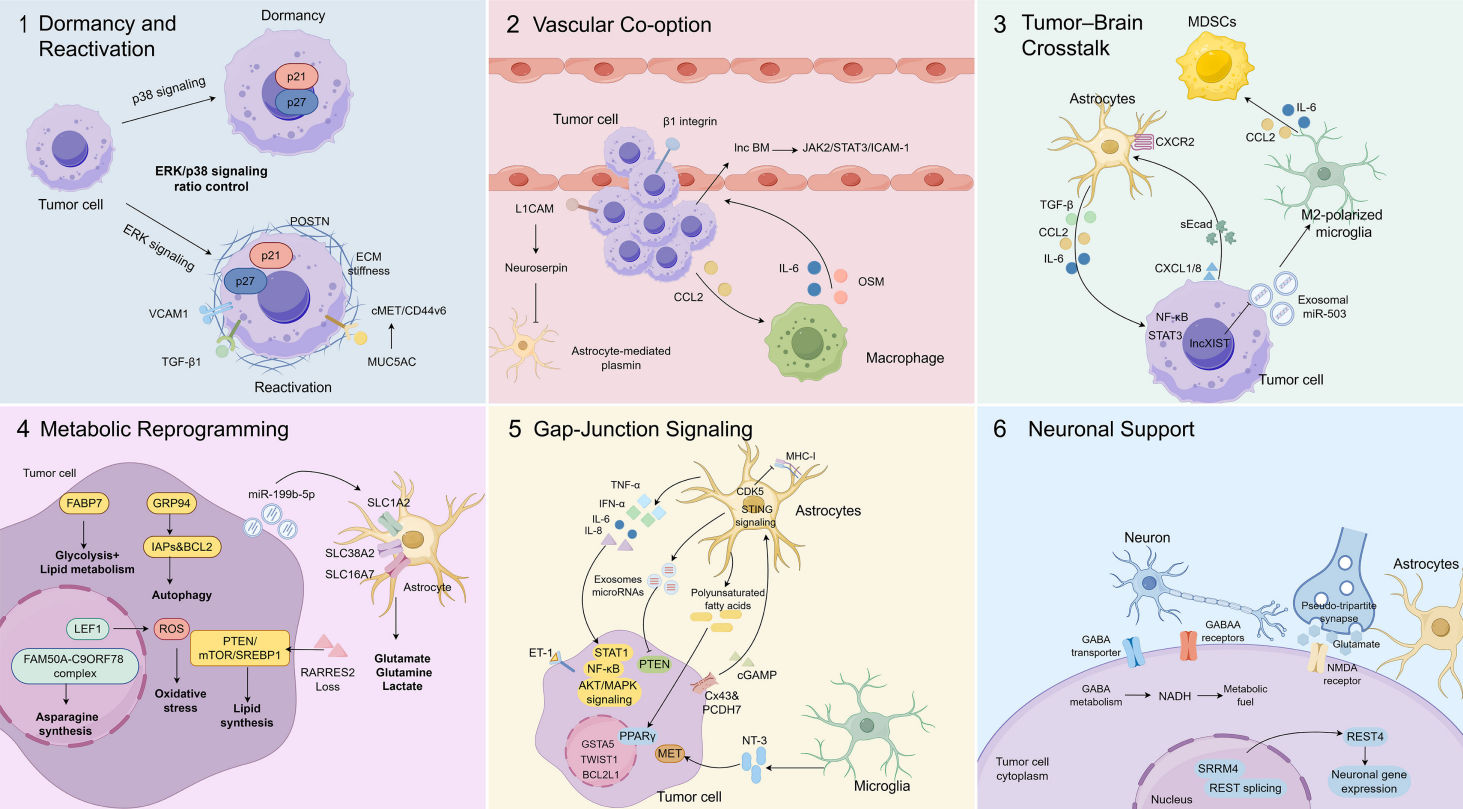
Molecular mechanisms underlying breast cancer brain colonization and adaptation within the brain microenvironment. Following extravasation into the brain parenchyma, disseminated breast cancer cells undergo a series of adaptive processes that enable metastatic colonization (1). Dormancy and reactivation: Disseminated tumor cells may initially enter a dormant state regulated by the ERK/p38 signaling balance. Microenvironmental cues including VCAM1, TGF-β1, POSTN, ECM stiffness, and MUC5AC-cMET/CD44v6 signaling promote tumor cell reactivation and proliferation (2). Vascular co-option: Metastatic cells exploit existing brain vasculature through β1 integrin- and L1CAM-mediated adhesion. Tumor-derived CCL2 recruits macrophages that secrete IL-6 and OSM, which activate the lnc-BM-JAK2/STAT3/ICAM-1 signaling axis to support vascular co-option and metastatic survival (3). Tumor-brain microenvironment crosstalk: Astrocytes and microglia undergo tumor-induced functional reprogramming. Cytokines such as IL-6, TGF-β and CCL2 activate STAT3 and NF-κB signaling, while exosomal miR-503 promote M2-like microglial polarization and immune suppression. M2-polarized microglia secrete IL-6 and CCL2 to recruit M-MDSCs, thereby sustaining an immunosuppressive microenvironment. In addition, tumor-derived soluble E-cadherin (sEcad) activates astrocytes through the CXCL1/8-CXCR2 signaling axis, reinforcing a pro-tumor feedback loop (4). Metabolic reprogramming: Metastatic breast cancer cells undergo metabolic reprogramming to adapt to the brain microenvironment. FABP7 enhances fatty acid utilization and glycolysis, whereas GRP94 upregulation promotes autophagy through increased IAPs and BCL2 expression. The FAM50A-C9ORF78 complex drives asparagine synthesis via ASNS, while LEF1-mediated glutathione production protects tumor cells from ROS-induced oxidative stress. Tumor-derived EV-miR-199b-5p disrupts neuron-astrocyte metabolic coupling by targeting SLC transporters (SLC1A2, SLC38A2 and SLC16A7), leading to extracellular accumulation of glutamate, glutamine and lactate. In addition, RARRES2 deficiency activates the PTEN-mTOR-SREBP1 pathway to promote lipid metabolic remodeling and tumor survival (5). Gap-junction signaling: Communication between tumor cells and astrocytes through Cx43-PCDH7 gap junctions enables cGAMP transfer and activation of the STING pathway in astrocytes, leading to IFN-α and TNF-α release and activation of STAT1/NF-κB signaling in tumor cells. Gap-junction signaling also induces IL-6/IL-8-ET-1 signaling that activates the AKT/MAPK pathway and confers therapy resistance. Astrocyte-derived exosomes silence PTEN, whereas polyunsaturated fatty acids activate PPARγ signaling to promote tumor proliferation. In addition, astrocytic CDK5 suppresses MHC-I presentation to facilitate immune evasion, while microglia support tumor survival through NT-3 mediated MET (6). Neuronal support: Metastatic cells acquire neuronal-like properties by expressing GABAA receptors and GABA transporters, enabling GABA metabolism to generate NADH as an alternative energy source. Tumor cells also form pseudo-tripartite synapses with glutamatergic neurons via NMDA receptor signaling, while SRRM4-mediated REST alternative splicing promotes neuronal gene programs that facilitate metabolic adaptation and metastatic persistence.

Together, colonization of BCBMs requires a coordinated sequence of events: survival through dormancy or dormancy escape, vascular co-option to secure a nutrient supply, and profound metabolic reprogramming to adapt to the brain microenvironment (**Figure 3**). A deeper understanding of these mechanisms will be critical for identifying therapeutic strategies to prevent colonization and improve outcomes for patients with BCBM.

## Tumor-brain crosstalk in BCBM

### Immunomodulation and immune evasion in the brain metastatic niche

The immune landscape of BCBM has historically been described as immune cold, reflecting low levels of lymphocyte infiltration and limited responsiveness to immune checkpoint blockade. However, this paradigm is increasingly recognized as incomplete. Rather than representing a passive consequence of immune privilege, the brain metastatic niche is now understood as an actively immunosuppressed microenvironment shaped by tumor–stroma interactions, myeloid reprogramming, and metabolic constraints that collectively restrain antitumor immunity.

Within this context, resident glial populations, including microglia and astrocytes, undergo tumor-induced functional reprogramming that actively constrains antitumor immunity. Microglia, the resident macrophages of the CNS, can polarize into an M1-like phenotype that supports leukocyte extravasation or into an M2-like phenotype that promotes angiogenesis and immune suppression. In BCBM, M2-polarized microglia secrete IL-6 and CCL2, activating STAT3 signaling and recruiting myeloid-derived suppressor cells (M-MDSCs), thereby sustaining an immunosuppressive niche (90, 91).

Tumor-intrinsic alterations further reinforce this suppressive circuitry. Downregulation of the long non-coding RNA XIST in tumor cells enhances the release of exosomal miR-503, which further induces M2 polarization and inhibits T-cell proliferation through the production of suppressive cytokines (92). Astrocytes similarly shift toward a tumor-supportive phenotype. Upon activation, they secrete cytokines such as IL-6, TGF-β, and CCL2, driving tumor migration and invasion while amplifying STAT3 and nuclear factor kappa-B (NF-κB) signaling (93, 94). Soluble E-cadherin (sEcad) from cancer cells engages the C-X-C motif chemokine ligand 1/8 (CXCL1/8)/C-X-C motif chemokine receptor 2 (CXCR2) axis to activate NF-κB in astrocytes, reinforcing this pro-tumor feedback loop (95). Collectively, these coordinated glial-tumor interactions establish an actively immunosuppressed ecosystem that shields metastatic cells from immune-mediated elimination rather than merely permitting immune escape.

### Therapy resistance and tumor survival

Astrocytes and microglia enhance tumor survival through both direct contact and paracrine communication. Gap junctions composed of connexin 43 (Cx43) and protocadherin 7 (PCDH7) form functional bridges between tumor cells and astrocytes, enabling the transfer of cyclic guanosine monophosphate-adenosine monophosphate (cGAMP) and activation of the stimulator of interferon genes (STING) pathway in astrocytes. The resulting release of interferon-α (IFN-α) and tumor necrosis factor-α (TNF-α) activates signal transducer and activator of transcription 1 (STAT1) and NF-κB in tumor cells, promoting chemoresistance and growth (96). Additional gap-junction-mediated signaling upregulates IL-6 and interleukin-8 (IL-8), leading to endothelin-1 (ET-1) secretion and ET-receptor activation, which stimulate the AKT/mitogen-activated protein kinase (MAPK) pathway and confer paclitaxel resistance (97). This communication also triggers pro-survival genes such as glutathione S-transferase alpha 5 (GSTA5), BCL2 like 1 (BCL2L1), and twist family bHLH transcription factor 1 (TWIST1) (98). Beyond junctional signaling, astrocyte-derived exosomes transfer microRNAs that silence the tumor-suppressor PTEN (99). Astrocyte-released polyunsaturated fatty acids activate peroxisome proliferator activated receptor gamma (PPARγ) to enhance tumor proliferation (100), while elevated cyclin-dependent kinase 5 (CDK5) activity in astrocytes suppresses major histocompatibility complex I (MHC-I) presentation, enabling immune evasion (101). Microglia complement these effects by supporting invasion through the Wnt-dependent pathways (102) and by releasing neurotrophin-3 (NT-3), which protects metastatic cells from cytotoxic stress and drives mesenchymal-to-epithelial transition (MET) via E-cadherin upregulation (103). The cumulative outcome is a resilient tumor population that thrives under therapeutic pressure.

### Metabolic and synaptic adaptation

Neurons provide additional metabolic and signaling support that facilitates tumor adaptation to the CNS milieu. BCBM cells acquire neuronal-like properties, expressing γ-Aminobutyric acid type A (GABAA) receptor and γ-aminobutyric acid (GABA) transporters (104). Uptake and catabolism of GABA generate nicotinamide adenine dinucleotide (NADH), serving as an alternative energy source that sustains proliferation. Concurrently, BC cells form pseudotripartite synapses with glutamatergic neurons, exploiting N-methyl-D-aspartate (NMDA) receptor activation to drive colonization and correlating with poor prognosis (105). The neural-specific splicing factor serine/arginine repetitive matrix 4 (SRRM4) promotes neuro-adaptation by redirecting RE1 silencing transcription factor (REST) splicing toward the REST4 isoform, which enhances expression of neurotransmission-related genes and supports tumor persistence under metabolic stress (106). These neuronal interactions underscore the capacity of metastatic cells to integrate into neural circuits and co-opt brain-specific signaling for metabolic flexibility and growth.

The interaction of BC cells with resident brain cells, including microglia, astrocytes, and neurons, is crucial for both the initiation and progression of brain metastases. Microglia and astrocytes, though initially capable of antitumor responses, are frequently reprogrammed to support tumor survival, immune evasion, and therapy resistance. Neurons provide metabolic and signaling inputs that further enhance tumor adaptation to the brain microenvironment. Together, these interactions create a permissive niche that sustains metastatic growth and presents multiple opportunities for therapeutic intervention.

Beyond descriptive characterization of individual stromal populations, the tumor-brain microenvironment is increasingly understood as a coordinated signaling network rather than a collection of isolated cellular interactions. Convergent activation of STAT3, NF-κB, and PI3K-AKT signaling pathways represents a central integrative axis through which astrocytes, microglia, endothelial cells, and infiltrating myeloid populations collectively sustain tumor survival and immune evasion. For example, astrocyte-derived IL-6 and CCL2 amplify STAT3-dependent transcriptional programs in both tumor and myeloid compartments, reinforcing an immunosuppressive feedback loop (91). These reciprocal interactions suggest that microenvironmental signaling actively shapes clonal selection, therapeutic resistance, and metabolic adaptation within the brain metastatic niche (**Figure 3**). Therapeutic strategies targeting single tumor-intrinsic pathways may therefore fail unless parallel stromal signaling circuits are simultaneously disrupted. These mechanistic convergences provide a conceptual framework for understanding the signal transduction pathways discussed below.

## Signal transduction pathways involved in brain metastasis

**Figure 4:**
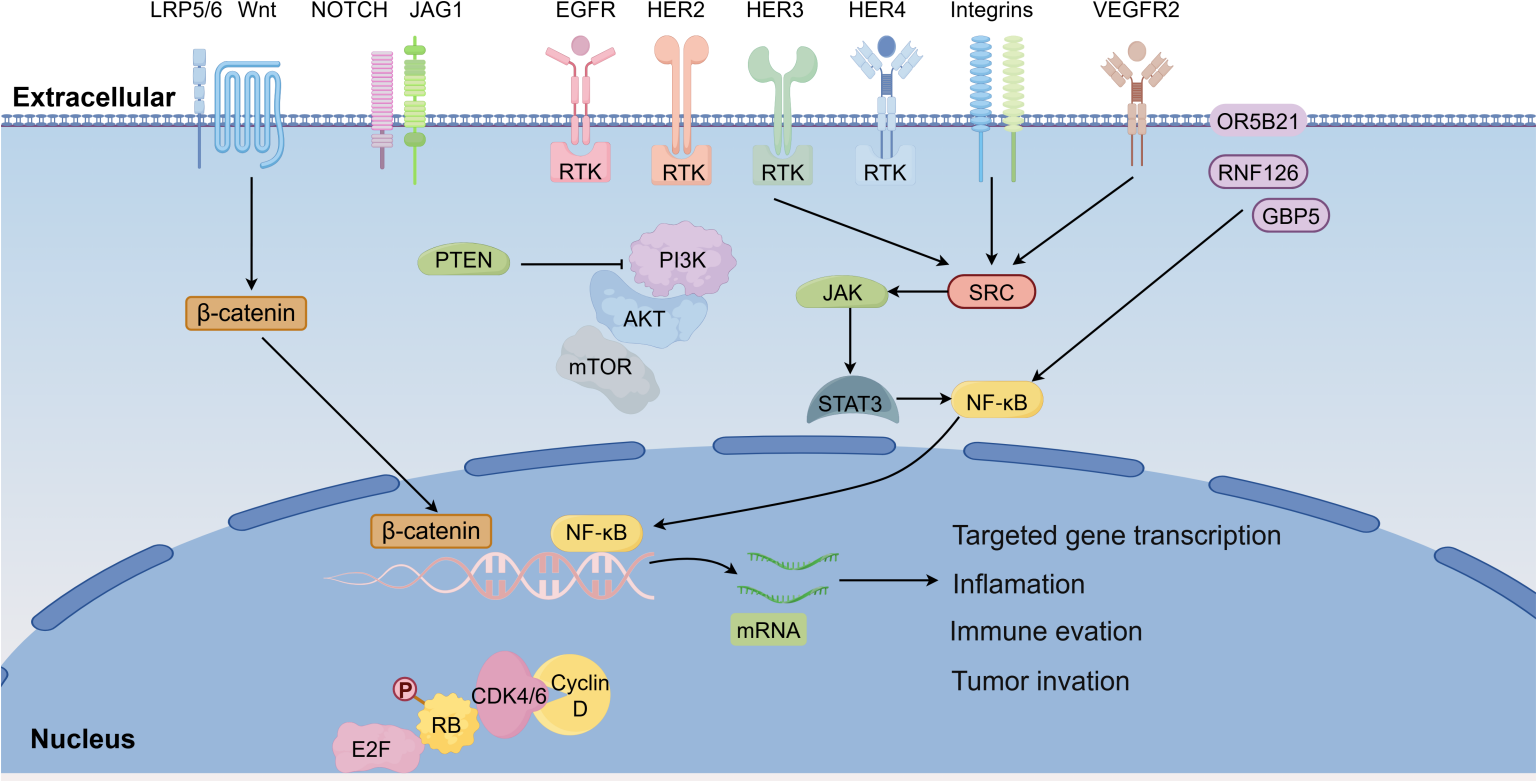
Integrated signaling network governing BCBM. BCBM is driven by a coordinated signaling network integrating RTKs activation, intracellular signaling cascades, and transcriptional programs that promote invasion, immune evasion, and metastatic colonization. Upstream membrane receptors, including EGFR, HER2, HER3, HER4, integrins, VEGFR2, and components of the Wnt and Notch pathways, initiate signaling events that activate downstream pathways. Canonical Wnt signaling stabilizes β-catenin to drive transcription of metastasis-associated genes, whereas RTKs activation stimulates the PI3K-AKT–mTOR pathway, which is negatively regulated by PTEN and promotes tumor cell survival and immune modulation. ERBB signaling further amplifies proliferative and survival pathways. SRC functions as a central signaling hub linking RTKs and integrins to cytoskeletal remodeling, invasion, and vascular permeability. Downstream activation of JAK-STAT3 and NF-κB signaling drives transcriptional programs associated with inflammation, immune suppression, and metastatic progression. Additional regulators including OR5B21, RNF126, and GBP5 further enhance NF-κB activity. In parallel, dysregulation of the cyclin D-CDK4/6-RB-E2F axis promotes cell-cycle progression and proliferative expansion of metastatic cells.

BCBM is governed by a complex network of signaling pathways that regulate tumor cell invasion, adaptation, immune evasion, and resistance to therapy. Among these, Wnt/Notch, PI3K/AKT/mTOR, ERBB, STAT3, Cyclin D-CDK4/6-RB-mediated cell cycle control, SRC, and NF-κB pathways have been most prominently implicated (**Figure 4**). Understanding how these molecular signals shape the brain metastatic niche provides opportunities for targeted therapeutic strategies.

### Wnt and Notch signaling pathway

The Wnt pathway can be activated through three distinct mechanisms: the canonical β-catenin-dependent canonical pathway, the planar cell polarity (PCP) pathway, and the Wnt/Ca²^+^ pathway. Activation of non-canonical Wnt signaling has been associated with the invasive behavior of basal-like BC subtypes, which are at high risk of developing brain metastases (107, 108).

The Notch pathway comprises four receptors (NOTCH1 through NOTCH4) and two groups of ligands: the Jagged family members (JAG1 and JAG2) and the Delta-like proteins (DLL1, DLL3, DLL4). Elevated JAG2 expression activates Notch signaling, promoting tumor cell migration and invasion, thereby contributing to CNS metastasis (109). Interleukin-1β (IL-1β), highly expressed in brain metastatic cells, upregulates JAG1 expression in astrocytes, further enhancing Notch signaling (110). In addition, notch receptor 3 (NOTCH3) has been shown to increase the invasive potential of TNBC cells derived from CNS metastases (111). Together, these findings implicate both Wnt and Notch as important drivers of metastatic dissemination and colonization.

### PI3K/AKT/mTOR signaling pathway and PTEN

The PI3K/AKT/mTOR signaling pathway plays a pivotal role in the development and progression of BCBM (112). Alterations in the phosphatidylinositol-4,5-bisphosphate 3-kinase catalytic subunit alpha (PIK3CA) gene, which encodes the p110α catalytic subunit of PI3K, are present in approximately 22% of BCBM cases, making it the second most frequently mutated gene after TP53 (113). Aberrant activation of this pathway induces immune-modulatory genes including programmed cell death ligand 1 (PD-L1), colonystimulating factor 1 (CSF1), colony-stimulating factor 1 receptor (CSF1R), and cytotoxic T lymphocyte- associated protein 4 (CTLA4) in both tumor cells and microglia. Inhibition of PI3K/AKT/mTOR signaling suppresses these genes and reduces the invasive capacity of metastatic cells (114).

The tumor suppressor PTEN counteracts PI3K/AKT signaling by dephosphorylating PIP2 and PIP3. Loss of PTEN prevents this regulatory step, resulting in sustained activation of the cascade. PTEN deficiency is observed more frequently in TNBC than in HR-positive/HER2-negative or HER2-positive subtypes (115, 116), and correlates with poor overall survival (OS) in TNBC patients who develop brain metastases (117). PTEN expression is consistently reduced in CNS lesions compared with primary tumors, with higher mutation frequencies observed in brain metastases (118). Notably, PTEN loss appears reversible. Specifically, tumor cells may downregulate PTEN within the brain microenvironment but restore its expression outside the CNS, which is influenced by astrocyte-derived microRNAs. Loss of PTEN also increases secretion of CCL2, which shapes a pro-metastatic microenvironment (99).

### ERBB signaling pathway

The ERBB family of receptor tyrosine kinases (RTKs) comprises four members: EGFR (also known as ERBB1 or HER1), ERBB2 (HER2), ERBB3 (HER3), and ERBB4 (HER4) (119). Among these, EGFR, HER2, and ERBB3 are strongly implicated in BCBM. EGFR mutations occur more frequently in brain metastases than in primary tumors or other distant metastases, particularly in TNBC patients, highlighting their elevated risk (116).

HER2 overexpression, driven largely by gene amplification and constitutive activation (120), significantly enhances brain colonization. Experimental introduction of HER2 into the brain-seeking MDA-MB-231-BR BC cell line markedly increased metastatic growth in the brain (121). The tumor suppressor forkhead box P3 (FOXP3) normally restrains HER2 expression; thus, FOXP3 loss promotes HER2 overexpression and metastatic progression (122).

ERBB3 is frequently co-expressed with HER2 in BC (123). Enhanced ERBB3 activity confers resistance to PI3K inhibitors in tumors with PIK3CA mutations and/or HER2 amplification. Inhibition of ERBB3 restores PI3K inhibitor sensitivity, underscoring its role in PI3K–AKT pathway activation and CNS metastatic progression (124).

### SRC signaling pathway

SRC is a prototypical non receptor tyrosine kinase and a central member of the Src family kinases, whose activity is tightly controlled by domain mediated autoinhibition and phosphorylation dependent conformational switching. Structurally, Src family kinases share conserved SH4, SH3, SH2, and kinase domains, with catalytic output regulated by reciprocal phosphorylation at the C terminal inhibitory tyrosine and the activation loop tyrosine, enabling rapid toggling between repressed and active states (125). Although activating mutations in SRC are uncommon, aberrant activation of Src family kinases is frequently observed across human cancers and has been linked to aggressive behavior and metastatic competence (126). Functionally, SRC acts as a signaling integrator downstream of RTKs, integrins, and other upstream inputs, coordinating proliferative, migratory, and survival programs in response to microenvironmental cues (126).

In tumor progression, SRC signaling regulates processes central to metastatic dissemination, including cytoskeletal remodeling, focal adhesion turnover, and cell motility. Src family kinases cooperate with focal adhesion kinase and scaffold proteins such as paxillin to control adhesion dynamics and invasion, while also engaging downstream pathways including MAPK and STAT signaling that support tumor cell survival and stress tolerance (127, 128). In BCBM, SRC signaling is mechanistically relevant because it bridges tumor cell invasiveness with endothelial barrier regulation at the BBB. VEGFR2 activation can trigger c-Src signaling at endothelial junctions and drive vascular permeability through junctional remodeling involving vascular endothelial cadherin complexes, providing a vascular mechanism that can facilitate tumor cell extravasation (129). Consistent with this tumor endothelium interface model, contemporary therapeutic reviews of BCBM identify Src family kinase activity as a rational targetable node implicated in invasion and brain metastatic progression, supporting the concept that sustained SRC centered signaling can contribute to both intracranial seeding and lesion expansion (130). Collectively, these observations position SRC signaling as a central hub that integrates tumor intrinsic motility and adaptive survival with microenvironment driven vascular permissiveness, providing a mechanistic rationale for targeting SRC associated networks in BCBM.

### Cell cycle regulation signaling pathway

Dysregulated cell cycle control is a hallmark of metastatic BC and epresents a central mechanism underpinning metastatic fitness. Cellular proliferation requires orderly progression through the G0/G1, S, G2, and M phases of the cell cycle, a process governed by cyclin-dependent kinases (CDKs) in complex with their cyclin partners. CDK activity is tightly regulated by mitogenic stimulation and restrained by checkpoint activation in response to genomic stress, ensuring coordinated DNA replication and mitotic fidelity (131). Mitogenic signaling promotes formation of cyclin-CDK complexes that drive the G1-to-S phase transition primarily through phosphorylation and functional inactivation of the retinoblastoma protein (RB). Loss of RB-mediated repression enables activation of E2F transcription factors, which orchestrate transcriptional programs required for DNA synthesis, chromatin remodeling, chromosome segregation, and mitotic spindle checkpoint integrity. The cyclin D-CDK4/6-RB axis therefore serves as a pivotal gatekeeper of proliferative commitment, coupling extracellular growth cues to sustained cell cycle progression (131, 132).

Pharmacological inhibition of CDK4/6 delays RB phosphorylation and attenuates E2F-driven transcription, thereby restricting S-phase entry and limiting proliferative expansion (132, 133). These findings underscore the centrality of this checkpoint in tumor growth control. In the context of brain metastasis, tumor cells encounter unique selective pressures, including metabolic constraint, limited trophic support, and therapeutic barriers imposed by the BBB. Under such conditions, sustained proliferative signaling is not merely a byproduct of oncogenic activation but becomes an essential requirement for successful intracranial outgrowth (134).

### STAT3 signaling pathway

The Janus kinase (JAK)-signal transducer and activator of transcription (STAT) pathway is a key signaling cascade activated by cytokine, and phosphorylated STAT3 (pSTAT3) has emerged as a hallmark of reactive astrocytes in brain metastases (135). These pSTAT3-positive astrocytes promote metastasis by suppressing CD8^+^ T cell activity through upregulation of immunosuppressive mediators including PD-L1, vascular endothelial growth factor-A (VEGF-A), lipocalin-2 (LCN2), and tissue inhibitor of metalloproteinases-1 (TIMP-1) (135). Similar findings in primary brain tumors show high STAT3 and PD-L1 expression driving an immunosuppressive cytokine milieu enriched in IL-10 and TGF-β (136).

STAT3 signaling also mediates astrocyte-microglia interactions. Astrocyte-derived migration inhibitory factor (MIF) binds CD74 on microglia, activating them via the MIF/CD74 axis. This leads to NF-κB dependent upregulation of midkine, a growth factor that accelerates metastatic progression (135). Additionally, STAT3-positive astrocytes protect cancer cells from chemotherapy by inducing survival gene expression through gap junction communication.

### NF-κB signaling pathway

The NF-κB pathway serves as a central regulator of inflammation responses, tumor cell invasion, and therapy resistance in BC. Upon activation, NF-κB translocates to the nucleus and drives transcription of genes involved in adhesion, migration, and invasion (137). In BCBM, NF-κB activation underpins immune evasion, enhances survival, and promotes colonization (138).

Several mediators activate NF-κB in BCBM. Olfactory receptor family 5 subfamily B member 21 (OR5B21) overexpression induces EMT through NF-κB, enhancing invasiveness (139). Elevated guanylate binding protein 5 (GBP5) expression in TNBC similarly activates NF-κB, promoting invasion and brain metastatic progression (140). FAK-mediated NF-κB activation strengthens tumor-brain microenvironment interactions, facilitating survival and growth (141). Furthermore, NF-κB contributes to therapy resistance. Specifically, ring finger protein 126 (RNF126) activates NF-κB to confer radioresistance, whereas its inhibition with dihydroartemisinin (DHA) restores radiosensitivity (142). Collectively, these findings highlight NF-κB as a central regulator of BCBM by integrating pathways that drive invasion, immune evasion, and therapy resistance. Targeting NF-κB signaling therefore represents a promising therapeutic avenue in the management of brain metastases.

Collectively, multiple oncogenic pathways have been implicated in BCBM, no single signaling axis uniformly governs intracranial progression. Pathway activation is subtype-dependent and shaped by prior systemic therapy, clonal evolution, and microenvironmental selection pressures. Furthermore, many mechanistic insights derive from preclinical models, and clinical translation remains limited by pharmacokinetic barriers and interlesional heterogeneity. Therefore, a balanced interpretation requires recognition of pathway redundancy and adaptive feedback rather than attributing brain metastasis to isolated signaling drivers. Importantly, biological plausibility does not equate to clinical actionability. Among the pathways implicated in BCBM, HER2-directed therapy currently represents the most convincingly translated paradigm, supported by randomized phase III evidence demonstrating meaningful intracranial benefit in active disease. In contrast, although PI3K/AKT/mTOR alterations are highly prevalent and mechanistically compelling, clinical data remain largely confined to early-phase or non-randomized studies without definitive CNS-specific endpoints. These discrepancies underscore a critical translational gap: pathway dependency alone is insufficient for therapeutic success. Effective clinical translation requires adequate CNS drug exposure, mitigation of adaptive signaling redundancy, and prospective trials explicitly powered for intracranial outcomes.

## Clinical advances in the management of BCBM

The management of BCBM has undergone substantial evolution over the past decade, driven by parallel advances in locoregional therapies, systemic treatments, and molecular stratification. Historically, therapeutic strategies were largely palliative and focused on symptom relief through surgery or WBRT, reflecting limited intracranial efficacy of conventional systemic agents. However, improved understanding of BBB biology, tumor heterogeneity, and subtype-specific vulnerabilities has enabled a shift toward more durable and individualized treatment paradigms. Contemporary management of BCBM increasingly relies on the rational integration of neurosurgical resection, precision radiotherapy, and targeted or immune-based systemic therapies, with the dual goals of achieving sustained intracranial control and preserving neurocognitive function and quality of life.

Importantly, clinical decision-making in BCBM is no longer guided solely by lesion number, but instead incorporates cumulative tumor volume, molecular subtype, extracranial disease status, and anticipated responsiveness to systemic therapy. Advances in stereotactic radiosurgery (SRS), hippocampal-sparing radiotherapy, antibody-drug conjugates (ADCs), BBB-penetrant kinase inhibitors, and emerging immunotherapeutic combinations have collectively expanded treatment options for patients previously considered to have limited prognosis. As a result, BCBM is increasingly managed as a chronic, biologically heterogeneous condition requiring longitudinal, multidisciplinary care. The following sections summarize recent clinical advances across locoregional and systemic modalities, highlighting how mechanistic insights and clinical trial evidence are reshaping therapeutic strategies for this challenging manifestation of BC.

### Locoregional treatments and therapeutic innovation in breast cancer brain metastases

Locoregional treatments remain the backbone of therapy for BCBM and play a pivotal role in achieving durable intracranial control, prolonging survival, and preserving neurological function and quality of life. Despite major advances in systemic therapies, most patients with BCBM still require neurosurgical and/or radiotherapeutic interventions during the course of their disease. In recent years, genuine innovations in radiotherapy techniques and treatment paradigms have reshaped the locoregional management of BCBM and enabled closer integration with systemic therapies.

#### Surgical resection of BCBM

Surgical resection remains an important component of multidisciplinary management when rapid symptom relief and durable local control are required, and its role should be framed by total intracranial tumor burden, lesion size and location, Karnofsky Performance Status (KPS), and the tempo of extracranial disease. Early randomized trials in patients with solitary brain metastases from solid tumors established a survival benefit of surgery combined with radiotherapy compared with radiotherapy alone. In a landmark randomized study, surgical resection followed by radiotherapy prolonged median OS from 15 weeks to 40 weeks and reduced local recurrence from 52% to 20%, supporting durable intracranial control in appropriately selected patients (143). Beyond solitary lesions, evidence is derived primarily from retrospective surgical series. Single-center analyses have reported the feasibility of resecting multiple symptomatic metastases in selected patients, with postoperative improvement in KPS documented in 71% of evaluable patients in one cohort (144), and in 42.6% of patients undergoing resection of two or more lesions in another contemporary series (145). These findings are consistent with retrospective volumetric analyses suggesting that total tumor burden, rather than lesion count alone, may have prognostic significance. Retrospective volumetric analyses further suggest that total tumor burden, rather than lesion count alone, may have prognostic significance, with cumulative tumor volume below 7 cm3 independently associated with longer survival after resection (146). High-level evidence from a multicenter, randomized, controlled phase 3 trial supports postoperative cavity-directed SRS. In NCCTG N107C, SRS resulted in significantly lower rates of cognitive deterioration at 6 months compared with whole brain radiotherapy (WBRT), 52% versus 85%, and prolonged cognitive deterioration-free survival, without a significant difference in median OS, 12.2 months versus 11.6 months (147). Collectively, these data support a stratified surgical strategy in BCBM that prioritizes patient selection based on functional status, cumulative tumor burden, and systemic disease control, with integration of focal radiotherapy to optimize intracranial control while preserving neurocognitive function.

#### Stereotactic radiosurgery and whole-brain radiotherapy

Despite continuous advances in systemic therapy, locoregional treatment remains the cornerstone of management for BCBM. It primarily relies on SRS and WBRT to achieve rapid and durable intracranial control. Historically, WBRT served as the standard of care for patients with multiple brain metastases. However, its well-documented association with profound neurocognitive decline has driven a paradigm shift away from WBRT and toward SRS. By delivering highly conformal, ablative doses of radiation to discrete intracranial lesions while sparing adjacent normal brain parenchyma, SRS achieves robust local tumor control with a substantially reduced risk of neurotoxicity. Mounting clinical evidence now supports the utility of SRS not only in patients with a limited burden of intracranial metastases, but also in carefully selected patients with multiple lesions, provided that the total intracranial tumor volume is deemed clinically acceptable. In a randomized controlled phase III trial, among patients with 1–3 brain metastases, the proportion of patients with cognitive deterioration at 3 months was significantly lower in the SRS-alone group than in the SRS plus WBRT group (63.5% vs. 91.7%), with no significant difference in OS. These findings provide level I evidence to support SRS as an alternative to the conventional approach of SRS combined with WBRT (148). In a prospective observational study, among patients with 2–4 versus 5–10 brain metastases treated with SRS alone, median OS was identical at 10.8 months in both groups, and SRS for 5–10 lesions was non-inferior to that for 2-4lesions (hazard ratio 0.97, 95% CI 0.81–1.18), with comparable rates of grade 3–4 treatment-related adverse events (2% vs 3%) (27). While these findings support careful expansion of SRS to patients with higher lesion counts, the absence of randomization and limited BC-specific stratification should be acknowledged. For patients with extensive intracranial lesions who still require WBRT, substantial innovations have also emerged in cognitive protection strategies. The RTOG 0614 randomized double-blind trial demonstrated that concurrent administration of memantine prolonged the time to cognitive decline, with a hazard ratio of 0.78; the probability of cognitive failure at 24 weeks decreased from 64.9% to 53.8% (149). In the phase III NRG Oncology CC001 trial, hippocampal avoidance (HA)-WBRT combined with memantine further reduced the risk of cognitive failure, yielding an adjusted hazard ratio of 0.74. Moreover, this regimen was associated with lower incidences of executive function deterioration at 4 months and learning-memory deterioration at 6 months, thereby improving symptomatic and functional outcomes without compromising intracranial progression-free survival (PFS) (150). These trials provide high-level evidence supporting cognitive-sparing WBRT approaches when WBRT remains indicated.

Beyond direct cytotoxicity, locoregional radiotherapy may also modulate the BBB, thereby influencing the intracranial exposure and efficacy of systemic therapies. In a pharmacokinetic study, the cerebrospinal fluid-to-plasma concentration ratio of gefitinib was 1.34% in patients with brain metastases, which was higher than the 0.36% observed in non-brain metastasis controls. This ratio increased with escalating radiation doses during concurrent WBRT, reaching 1.87% at a dose of 30 Gy, suggesting that radiotherapy can enhance BBB permeability and potentially improve the intracranial penetration of certain targeted agents (151). This biological rationale has propelled the tighter integration of SRS and systemic therapies. Particularly in HER2-positive BCBM, the combination of locoregional radiotherapy and small-molecule targeted agents has been employed to augment local control. However, such findings derive from small non-randomized cohorts and require cautious interpretation. Combination strategies integrating radiotherapy with targeted therapy or immunotherapy are supported primarily by retrospective analyses and early-phase studies. Retrospective studies have shown that concurrent administration of lapatinib with SRS reduced the 12-month risk of local failure from 15.1% to 5.7%, alongside a lower risk of radiation necrosis (152). Taking the phase I study of nivolumab combined with SRS as an example, among 12 patients with BCBM, the 12-month local control rate was 94% and the 12-month OS rate was 75%, with no radiation necrosis observed. Concomitant dynamic changes in immune-related biomarkers were noted, indicating the feasibility and potential synergism of combining radiotherapy with immune checkpoint inhibitors (ICIs) (153). While these findings are encouraging, they derive from non-randomized or early-phase studies and require validation in larger prospective trials incorporating predefined CNS-specific endpoints and active brain metastasis populations.

Locoregional therapy for BCBM is evolving from mere palliative intracranial debulking towards precision radiotherapy strategies oriented around neurocognitive preservation. By modulating the BBB and reshaping the immune microenvironment, it provides both mechanistic and clinical evidence to underpin the rational combination of radiotherapy with targeted therapies and ICIs. Future research should prioritize intracranial-specific endpoints to further clarify the optimal sequencing of radiotherapy and systemic therapies, as well as risk management strategies, with the aim of maximizing intracranial control while preserving neurological function and quality of life to the greatest extent possible.

### Molecularly targeted therapy

#### HER2-positve inhibitors

HER2 overexpression occurs in approximately 20-25% of BC (154). As a RTK localized on the plasma membrane, HER2 enhances extracellular signaling to drive cell survival and proliferation through multiple downstream effectors (155). In a pivotal randomized phase III trial, trastuzumab, the first HER2-targeted monoclonal antibody, significantly improved PFS when combined with chemotherapy regimens such as anthracycline with cyclophosphamide, paclitaxel, or other standard treatments, compared to chemotherapy alone (156). Pertuzumab, which binds a different epitope of HER2, further improved outcomes when added to trastuzumab and docetaxel. In the CLEOPATRA trial, this triplet increased OS by 15.7 months, reaching 56.5 months, establishing it as a first-line standard of care (157). However, it is important to note that these landmark phase III trials were not designed specifically for patients with active brain metastases, and CNS-specific endpoints were not primary outcomes. Evidence supporting the role of trastuzumab in patients who subsequently develop brain metastases derives largely from retrospective analyses, which suggest improved OS through sustained systemic disease control rather than direct intracranial activity (158–160). Thus, while HER2-directed antibodies provide high-level evidence in systemic disease, their intracranial efficacy is inferred rather than prospectively established in early trials.

Small-molecule tyrosine kinase inhibitors (TKIs) provide an advantage by penetrating the BBB. Lapatinib, a dual EGFR/HER2 inhibitor, demonstrated CNS activity in the single-arm phase II LANDSCAPE trail, where lapatinib plus capecitabine produced objective intracranial responses in 65.9% of patients with untreated HER2-positive BCBM (161). Although encouraging, this study lacked randomization and comparator arms. Similarly, the phase III NALA trial demonstrated improved PFS with neratinib plus capecitabine compared with lapatinib plus capecitabine in heavily pretreated metastatic disease (162); however, intracranial efficacy analyses were exploratory and limited to subgroup evaluation. More definitive intracranial evidence emerged from the HER2CLIMB trial. This randomized phase III study prospectively enrolled patients with both stable and active brain metastases and demonstrated that the addition of tucatinib to trastuzumab and capecitabine significantly improved CNS progression-free survival and OS in patients with baseline brain metastases (163). Earlier phase Ib data had already suggested CNS activity (164), but HER2CLIMB provides the highest level of randomized evidence supporting a HER2-directed regimen with clinically meaningful intracranial efficacy. Nevertheless, even in this trial, intracranial endpoints were secondary analyses, underscoring the need for future studies specifically designed with CNS-focused primary outcomes.

#### Antibody-drug conjugate

Systemic therapy for BCBM has long been hampered by suboptimal therapeutic efficacy, which is largely attributable to two key factors: the BBB of the CNS restricts intracranial drug exposure, and most pivotal clinical trials have systematically excluded patients with active brain metastases, resulting in a paucity of high-quality evidence-based data. The emergence of ADCs has ushered in a novel therapeutic paradigm for this challenging disease setting. Ado-trastuzumab emtansine (T-DM1) demonstrated OS superiority over lapatinib plus capecitabine in the randomized phase III EMILIA trial in previously treated HER2-positive metastatic BC. However, patients with brain metastases were not prospectively stratified, and intracranial endpoints were not primary outcomes. In a retrospective exploratory subgroup analysis of EMILIA, 45 patients with baseline brain metastases received T-DM1 and 50 received lapatinib plus capecitabine (165). Within this subgroup, median OS was 26.8 months in the T-DM1 arm versus 12.9 months in the control arm. While these data suggest clinically meaningful benefit, the retrospective nature and limited sample size warrant cautious interpretation. Additional real-world analyses have reported CNS objective response rates of approximately 24.5% with T-DM1 (166), but these findings derive from non-randomized cohorts and reflect heterogeneous populations with stable and active lesions.

More recently, trastuzumab deruxtecan (T-DXd, DS8201), a next-generation ADCs, has shown promising intracranial activity and potential survival benefits in patients with stable brain metastasis. TUXEDO-1 was a single-arm phase II study that exclusively enrolled patients with HER2-positive BC and untreated or progressive brain metastases. The investigator-assessed best intracranial objective response rate following trastuzumab deruxtecan administration was 73.3%, including a complete response rate of 13.3% and a partial response rate of 60.0%, with stable neurocognitive function and quality of life preserved throughout the follow-up period (167). Importantly, this study focused specifically on active brain metastases and demonstrated preserved neurocognitive function, providing the first prospective signal that ADC therapy can achieve substantial intracranial responses in progressive disease. However, the absence of a comparator arm limits conclusions regarding relative efficacy. DESTINY-Breast12, a prospective phase IIIb/IV study, was the first large prospective trial to incorporate a dedicated brain metastasis cohort in its design (168). Among patients with baseline CNS involvement, the 12-month CNS progression-free survival rate was 58.9%, and benefit was observed in both stable and active brain metastasis subgroups. These findings provide the most robust prospective evidence to date supporting durable intracranial activity of T-DXd. Nevertheless, interstitial lung disease occurred in 16% of patients in the brain metastasis cohort, including 3% grade 3 or higher events, underscoring the need to balance efficacy with vigilant toxicity monitoring.

Collectively, ADCs illustrate the evolution from retrospective subgroup signals toward prospective CNS-inclusive trial design. However, even in recent studies, intracranial endpoints remain secondary or cohort-based rather than primary outcomes, highlighting the continued need for randomized trials specifically powered for active brain metastases.

#### CDK4/6 inhibitors

CDKs control the progression of the cell cycle by partnering with cyclins to drive transitions between phases. Specifically, CDK4 and CDK6 control the transition from the G0/G1 phase to the S phase, thereby promoting DNA synthesis and proliferation (169). In HR-positive metastatic BC, selective CDK4/6 inhibitors, including palbociclib, ribociclib, abemaciclib, and dalpiciclib, are established standard-of-care therapies in combination with endocrine treatment, based on multiple phase III randomized trials demonstrating improvements in PFS and OS (170–173). However, pivotal registration trials largely excluded patients with active brain metastases, limiting high-level evidence for intracranial efficacy. Among available agents, abemaciclib has demonstrated measurable CNS penetration and has been prospectively evaluated in a dedicated phase II study of patients with HR -positive BCBM (174). In this single-arm trial, abemaciclib achieved detectable drug concentrations within resected brain metastasis tissue and produced an intracranial clinical benefit rate of 24% in heavily pretreated patients. Although objective response rates were modest, these findings provide proof of biological CNS activity. Nevertheless, absence of randomized comparison and limited sample size underscore that the intracranial benefit of CDK4/6 inhibition remains investigational rather than definitively established.

#### PI3K/AKT/mTOR inhibitors

Alterations in the PI3K/AKT/mTOR signaling cascade have been reported in 43-75% of BCBM cases, highlighting this signaling axis as a potential therapeutic target (117, 175, 176). Much of the mechanistic evidence derives from preclinical studies. In xenograft models of PIK3CA-mutant brain metastases, the pan-AKT inhibitor GDC-0068 demonstrated pro-apoptotic effects and improved survival, providing proof-of-concept that pathway inhibition can impair intracranial tumor growth (177). Similarly, the dual PI3K-mTOR inhibitor GDC-0084 achieved measurable antitumor activity in *in vitro* and *in vivo* brain metastasis models and demonstrated BBB penetration in experimental systems (178). However, these findings remain preclinical and have not yet been validated in randomized intracranial clinical trials.

In systemic metastatic BC, mTOR inhibition with everolimus is supported by randomized phase III evidence. The BOLERO-2 trial demonstrated that everolimus combined with exemestane significantly improved PFS in HR-positive advanced disease (179), and BOLERO-3 showed benefit in trastuzumab-resistant HER2-positive metastatic BC (180). Importantly, these trials were not designed specifically for patients with active brain metastases and did not include CNS-specific primary endpoints. Clinical evidence directly addressing intracranial efficacy of PI3K-AKT-mTOR pathway inhibition remains limited. In a phase Ib/II single-arm study evaluating everolimus in combination with lapatinib and capecitabine for HER2-positive brain metastases, a CNS objective response rate of 27% at 12 weeks and a median PFS of 6.2 months were reported (181). While these data suggest biological activity in the CNS, the absence of randomization, modest sample size, and heavily pretreated population limit definitive conclusions regarding therapeutic benefit. Collectively, although PI3K-AKT-mTOR alterations are common in BCBM and represent compelling biological targets, current clinical evidence for intracranial efficacy is largely derived from early-phase or non-randomized studies. Translation of pathway dependency into durable CNS benefit remains constrained by pharmacokinetic challenges and the lack of prospective trials incorporating predefined CNS-specific endpoints.

#### PARP inhibitors

Poly (ADP-ribose) polymerase (PARP) inhibitors, including olaparib, rucaparib, niraparib, veliparib, and talazoparib, have demonstrated efficacy in germline BRCA1/2-mutated metastatic BC and have received regulatory approval based on randomized phase III trials (182, 183). In the OlympiAD trial, olaparib significantly improved PFS compared with standard chemotherapy in patients with germline BRCA-mutated metastatic disease (183). Similarly, talazoparib improved PFS in the EMBRACA trial (182). These pivotal studies established PARP inhibition as a standard systemic therapy in selected populations but largely excluded patients with active or untreated brain metastases, and intracranial endpoints were not primary outcomes. BRCA1 and BRCA2 play essential roles in homologous recombination repair (HRR), checkpoint control of the cell cycle, and transcriptional regulation (184). As core components of the DNA repair machinery, BRCA protein help resolve double-stranded DNA breaks. Clinical studies indicate that patients with germline BRCA1/2 are more likely to develop CNS metastases than non-carriers (185, 186). Genomic analyses further demonstrate that BCBM exhibit higher homologous recombination deficiency (HRD) scores than matched primary tumors, with more than half of tested brain lesions classified as HRD-positive (187). These findings support the biological plausibility of PARP sensitivity in intracranial disease. However, clinical evidence specifically demonstrating robust intracranial responses remains limited. Most approved PARP inhibitors, including olaparib, rucaparib, and talazoparib, exhibit limited penetration across the intact BBB, and prospective CNS-focused trials are lacking. While veliparib and emerging brain-penetrant PARP1-selective compounds are under investigation, definitive randomized data demonstrating durable intracranial efficacy are not yet available. Taken together, although HRD is enriched in BCBM and provides a compelling biological rationale for PARP inhibition, current clinical evidence supporting meaningful activity against active brain metastases remains preliminary. Effective therapeutic exploitation will likely require integration of genomic selection with agents capable of achieving sufficient CNS exposure.

### Immune checkpoint therapy

The immune checkpoint protein PD-L1, expressed on tumor cell surfaces, binds to programmed cell death-1 (PD-1) receptors on cytotoxic T lymphocytes to induce T cell exhaustion or apoptosis (188). ICIs have transformed treatment paradigms in multiple malignancies. In BC, however, robust efficacy has primarily been demonstrated in triple-negative disease. In metastatic TNBC, the randomized phase III IMpassion130 trial showed that atezolizumab combined with nab-paclitaxel improved PFS in PD-L1-positive tumors, although patients with untreated or symptomatic brain metastases were excluded and intracranial endpoints were not primary outcomes (189). The immune microenvironment of BCBM exhibits distinct immunobiological features. Brain metastases frequently demonstrate reduced immune infiltration relative to matched primary tumors and enrichment of immunosuppressive cell populations, including FOXP3^+^ regulatory T cells (Tregs) (190). Moreover, immune infiltration is generally lower in BCBM compared with matched primary tumors. Nonetheless, higher densities of tumor-infiltrating lymphocytes (TILs) within brain lesions have been associated with improved survival, suggesting that intracranial immune activation retains prognostic relevance. Clinical data specifically evaluating ICIs in active BCBM remain sparse. Most pivotal trials have excluded patients with untreated or progressing brain metastases, limiting extrapolation. Small phase II and non-randomized studies have reported signals of intracranial activity in selected patients, but sample sizes are limited and CNS endpoints are often secondary (191, 192). Consequently, current evidence does not establish immune checkpoint blockade as a uniformly effective strategy for active brain metastases. Rather than conceptualizing the brain solely as an immune-cold organ, emerging evidence supports a model of active immunosuppression characterized by myeloid cell recruitment, regulatory T-cell enrichment, and cytokine-mediated inhibition. Future therapeutic strategies will likely require rational combination approaches aimed at enhancing T-cell trafficking, overcoming local immunosuppressive circuits, and integrating radiotherapy or vascular modulation to optimize drug delivery within the CNS.

## Microenvironment-directed therapeutic strategies

Therapeutic progress in BCBM has been driven largely by tumor intrinsic targets, yet durable intracranial control remains limited by microenvironmental dependencies and pharmacologic barriers. The metastatic niche comprises reactive astrocytes, microglia and infiltrating macrophages, endothelial and perivascular stromal elements, and ECM networks that jointly sustain immune suppression, vascular remodeling, and metabolic adaptation. Microenvironment oriented interventions therefore provide a complementary therapeutic framework that aims to disable the permissive ecosystem required for brain colonization and outgrowth, rather than solely intensifying tumor cell cytotoxicity.

### Vascular targeting

Antiangiogenic therapy represents a rational microenvironment-directed strategy in BCBM, as angiogenesis is a fundamental driver of metastatic progression within the intracranial TME. Tumor cells secrete pro-angiogenic mediators, including VEGF and fibroblast growth factors, which stimulate endothelial proliferation, vascular remodeling, and the formation of aberrant neovasculature. These structurally and functionally abnormal vessels not only sustain tumor growth by supplying oxygen and nutrients but also contribute to peritumoral oedema and immune dysregulation (193, 194). However, in BCBM, vascular biology is further complicated by the heterogeneous integrity of the BTB. Experimental models have demonstrated substantial inter-lesional variability in BTB permeability, directly influencing intratumoral drug delivery and therapeutic response. In this context, inadequate vascular permeability may decouple molecular pathway dependency from clinical efficacy, even when tumor cells remain biologically targetable (51). This insight provides a mechanistic rationale for vascular-targeting approaches that aim not only to suppress angiogenic support but also to improve functional perfusion and optimize drug penetration. In a phase 2 trial enrolling patients with new or progressive brain metastases from BC carboplatin plus bevacizumab achieved a CNS objective response rate of 63%, with median PFS of 5.62 months and median OS of 14.10 months, suggesting that VEGF axis modulation can yield clinically meaningful intracranial activity in selected settings (195).

### Myeloid targeting

BCBM is increasingly understood as an actively immunosuppressed niche rather than a passive immune privileged site, with myeloid populations functioning as dominant suppressors of antitumor immunity. Reactive astrocytes and myeloid cells establish cytokine networks that restrain T cell activity and promote metastatic fitness. In a landmark mechanistic study, STAT3 activation in a subpopulation of reactive astrocytes was required for efficient brain metastasis, and high astrocytic STAT3 activity in patient samples correlated with inferior survival after intracranial metastasis diagnosis, supporting astrocyte centered immune suppression as a tractable vulnerability (135). Complementing this concept, depletion or functional reprogramming of CNS myeloid cells through colony stimulating factor 1 receptor (CSF1R) inhibition reduced brain metastasis burden in preclinical models, indicating that macrophage and microglia dependencies can be therapeutically exploited (196). Notably, a preclinical report further suggested that CSF1R inhibition could prevent and treat TNBC brain metastases in hematogenous models, strengthening the rationale for myeloid directed combinations in aggressive subtypes (197).

### Chemokine axis targeting

Chemokine signaling provides a mechanistic bridge between vascular extravasation, myeloid recruitment, and niche maintenance. Astrocyte derived CCL2 promotes BC cell transmigration across the BBB through the CCL2-CCR2 axis, establishing a direct tumor-astrocyte program that facilitates extravasation and subsequent colonization (198). Therapeutically, this axis has become an attractive target for rational immunotherapy combinations, supported by translational work showing that CCL2 pathway blockade can cooperate with immune based strategies to counteract immunosuppressive recruitment programs in brain metastasis models (94). In parallel, the CXCL12-CXCR4 axis contributes to organ directed migration and stromal support programs in BC, and remains a candidate pathway for intercepting brain tropism and metastatic seeding, particularly when integrated with immune modulation or vascular targeting (199).

### Extracellular vesicle targeting

Tumor derived EVs are increasingly recognized as microenvironmental engineers that condition the brain endothelium and stromal compartments before overt colonization. A seminal study demonstrated that exosomal integrin signatures determine organotropic metastasis by preferentially targeting resident cells at metastatic destinations, including brain endothelial cells, and by priming the pre metastatic niche (200). These insights support therapeutic strategies focused on blocking extracellular vesicle biogenesis, uptake, or cargo mediated signaling, with the goal of interrupting early niche priming and reducing metastatic seeding efficiency.

### Metabolic targeting

The brain niche imposes distinctive metabolic constraints that can be co-opted by tumor cells through adaptive rewiring and metabolite exchange with stromal cells. BCBM cells can acquire glucose independent growth supported by enhanced gluconeogenesis and oxidation of glutamine and branched chain amino acids, and suppression of fructose 1,6 bisphosphatase reduced viability and improved host survival in immunocompetent models (85). Complementary work in brain tropic models showed that metabolic diversity and plasticity among disseminated tumor cell states governs metastatic fitness, highlighting lactate utilization and redox control as determinants of intracranial persistence (201). These observations provide a mechanistic basis for targeting metabolic coupling and stress tolerance programs as adjuncts to receptor directed therapy, particularly where BTB heterogeneity limits uniform drug exposure.

## Stable and active brain metastases: implications for clinical decision-making and trial design

A central practical distinction in BCBM is whether intracranial disease is stable after prior local therapy or active, meaning newly diagnosed untreated lesions or radiographically progressing lesions despite prior surgery or radiotherapy. This distinction matters because stable BCBM has historically been overrepresented in systemic therapy trials, whereas active BCBM is the scenario in which rapid neurological decline, steroid dependence, and early need for salvage radiotherapy most often occur. Increasing inclusion of active BCBM in prospective studies has therefore become a key driver of recent therapeutic innovation, shifting the goal from simply controlling extracranial disease to achieving clinically meaningful intracranial responses that can defer or reduce the intensity of locoregional treatment and its neurotoxicity.

In HER2-positive disease, HER2CLIMB provides the highest level of evidence for the management of active brain metastases. In this randomized phase III trial, patients were prospectively stratified as having active or stable brain metastases at baseline. Among those with brain involvement, the addition of tucatinib to trastuzumab and capecitabine improved CNS progression-free survival from 4.2 months to 9.9 months, with a hazard ratio of 0.32, and improved OS from 12.0 months to 18.1 months, with a hazard ratio of 0.58. In patients with measurable intracranial disease, the intracranial objective response rate increased from 20.0% to 47.3% (202). These data establish a randomized benchmark for treating active intracranial disease rather than restricting evidence to clinically stable populations. Phase II studies enriched for progressive brain metastases further support the concept that brain-penetrant HER2 inhibition combined with chemotherapy can produce meaningful intracranial responses. In TBCRC 022, neratinib plus capecitabine achieved a composite CNS objective response rate of 49% in lapatinib-naive patients with measurable progressive brain metastases and a median PFS of 5.5 months (203). Although non-randomized, this study specifically addressed active disease and quantified intracranial endpoints prospectively. ADCs have further extended systemic treatment paradigms into active BCBM. In TUXEDO-1, trastuzumab deruxtecan produced high intracranial response rates in untreated or progressive brain metastases (168), supporting the feasibility of systemic therapy in carefully selected patients when immediate local intervention is not mandated. In contrast, cohorts dominated by previously treated and largely stable brain metastases have demonstrated more modest activity. In the phase IIIb KAMILLA study, which included 398 patients with baseline brain metastases, T-DM1 achieved a best intracranial objective response rate of 21.4% among patients with measurable lesions and a median progression-free survival of 5.5 months (204). Although clinically relevant, these outcomes reflect the heterogeneity of stable populations and the absence of randomized CNS-specific comparison.

Outside HER2-positive disease, prospective evidence for active BCBM remains limited. In HR-positive, HER2-negative disease, a dedicated phase II study of abemaciclib did not meet its primary endpoint but reported an intracranial clinical benefit rate of 24% and demonstrated drug concentrations in resected brain metastasis tissue exceeding *in vitro* inhibitory thresholds for CDK4/6 (174). These findings support biological activity but do not constitute definitive evidence of durable intracranial control. In metastatic TNBC, ASCENT largely limited enrollment to stable brain metastases and a *post hoc* subgroup analysis showed only a small numerical PFS improvement with sacituzumab govitecan compared with treatment of physician’s choice, 2.8 months versus 1.6 months, with similar OS, 7.0 months versus 7.5 months (205). These data underscore the persistent evidentiary gap between stable and actively progressing brain metastases and highlight the need for trials prospectively powered for intracranial endpoints in active disease.

## Liquid biopsy for detection and monitoring of BCBM

Emerging evidence indicates that liquid biopsy approaches may provide clinically meaningful tools for the detection and monitoring of BCBM, particularly in scenarios where repeated tissue sampling is impractical. Circulating tumor DNA (ctDNA) derived from cerebrospinal fluid demonstrates substantially higher sensitivity for detecting intracranial genomic alterations than plasma ctDNA, with concordance rates exceeding 80% when compared with matched brain metastasis tissue (206). In a prospective analysis of patients with brain metastases from solid tumors, cerebrospinal fluid ctDNA identified clinically actionable mutations that were undetectable in paired plasma samples, directly influencing therapeutic decision-making (207). Beyond detection, longitudinal ctDNA profiling has shown potential utility in monitoring intracranial treatment response and minimal residual disease, with dynamic changes in variant allele frequency preceding radiographic progression by several weeks (208). By contrast, CTCs are detected at low frequency in patients with isolated brain metastases, reflecting limited egress across the blood–brain barrier and restricting their current clinical applicability. Collectively, these findings support liquid biopsy, particularly cerebrospinal fluid ctDNA, as a promising adjunct to imaging for risk stratification, early relapse detection, and real-time assessment of intracranial disease dynamics in BCBM.

## Conclusion

BCBM remains a major clinical challenge, reflecting both the increasing survival of patients with advanced BC and the unique biological barriers of CNS. The pathogenesis of BCBM is a multistep process involving disruption of the BBB, colonization and metabolic adaptation within the brain parenchyma, and extensive crosstalk with resident brain cells, including astrocytes, microglia, and neurons. Key signaling pathways, such as Wnt/Notch, PI3K/AKT/mTOR, ERBB, STAT3, Cyclin D-CDK4/6-RB-mediated cell cycle control, SRC, and NF-κB pathways, cooperate to drive invasion, survival, immune evasion, and resistance, highlighting the complexity of metastatic adaptation. Recent therapeutic advances have improved outcomes but remain limited. HER2-directed monoclonal antibodies and BBB-permeable TKIs demonstrate intracranial efficacy in HER2-positive BCBM, whereas certain ADCs have shown activity across both HER2-positive and HER2-low disease. Targeted therapies, including CDK4/6 inhibitors for HR-positive disease, PI3K/AKT/mTOR blockade for pathway-altered tumors, and PARP inhibitors for BRCA-deficient subtypes, have expanded treatment options. Immunotherapy remains in early development but may achieve greater efficacy through rational combinations with SRS, PARP or PI3K inhibitors, or next-generation cellular and viral therapies.

Future progress depends on integrating molecular insights with therapeutic innovation. Advanced preclinical models, such as patient-derived xenografts, brain organoids, and BBB-on-chip systems, will clarify molecular dependencies and improve translational predictability (209–211). Single-cell and spatial multi-omics can map tumor heterogeneity and identify actionable vulnerabilities (212). Development of BBB-permeable drug conjugates and nanocarriers will enhance intracranial drug delivery while limiting systemic toxicity. Immunomodulatory strategies that aim to transform the immune-cold brain microenvironment into an immune-active niche, including checkpoint blockade, CAR-T cell therapy, and oncolytic virotherapy, hold significant therapeutic promise (213). Clinically, future trials must include CNS-specific endpoints, incorporate liquid biopsy and imaging biomarkers, and emphasize preservation of neurocognitive function alongside survival.

In summary, the treatment of BCBM requires a unified approach that combines molecular precision, improved CNS drug delivery, and immune reprogramming.
